# Readability and Comprehension of Anesthesia Informed Consent Forms in a Spanish County Hospital: An Observational Study

**DOI:** 10.3390/nursrep14020101

**Published:** 2024-05-24

**Authors:** José Manuel García-Álvarez, Alfonso García-Sánchez

**Affiliations:** 1 Health Sciences Program, Catholic University of Murcia (UCAM), Guadalupe, 30107 Murcia, Spain; 2 Faculty of Nursing, Catholic University of Murcia (UCAM), Guadalupe, 30107 Murcia, Spain; agarcia@ucam.edu

**Keywords:** informed consent forms, anesthesia, readability, comprehension, nursing

## Abstract

(1) Background: The wording of informed consent forms could hinder their comprehension and hinder patients’ autonomous choice. The objective of this study was to analyze the readability and comprehension of anesthesia informed consent forms in a Spanish county hospital. (2) Methods: Descriptive and cross-sectional study carried out on patients who were going to undergo anesthetic techniques. The readability of the forms was analyzed using the INFLESZ tool and their subjective comprehension using an ad hoc questionnaire. (3) Results: The analyzed forms presented a “somewhat difficult” legibility. A total of 44.2% of the patients decided not to read the form, mainly because they had previously undergone surgery with the same anesthetic technique. The language used in the forms was considered inadequate by 49.5% of the patients and 53.3% did not comprehend it in its entirety. A statistically significant negative correlation of age and INFLESZ readability score with the overall questionnaire score was found. A statistically significant association was observed as a function of age and educational level with the different criteria of the questionnaire. (4) Conclusions: The anesthesia informed consent forms presented low readability with limited comprehension. It would be necessary to improve their wording to favor comprehension and to guarantee patients’ freedom of choice.

## 1. Introduction

The bioethical principle of autonomy is based on the freedom of choice that allows patients to participate actively, together with healthcare professionals, in all aspects related to their health. This ethical and legal obligation is supported by the process of informed consent and is embodied in the reading and signing of an informed consent form for a given procedure [1,2]. In order for patients to be able to choose freely, the information provided in the informed consent forms must be truthful, sufficient, and supported by the existing scientific evidence. The wording of informed consent forms can be a major barrier to comprehension, hindering patients’ ability to make an autonomous choice. Therefore, these forms should be written in a clear and simple manner so that they can be adapted to the characteristics and needs of the specific patients for whom they are intended, allowing adequate comprehension to guarantee patients’ freedom of choice. The content of the informed consent forms used in Spain has been drafted by the national scientific societies of each specialty without taking into account the specific characteristics of different population groups [3,4,5].

Informed consent forms should be adequately structured to include all the information that patients may need to make a decision according to their needs and preferences [6,7,8,9,10]. Therefore, all sections of the informed consent forms considered necessary should be present and duly completed to ensure that they include all relevant and specific information that the patient may need to make an appropriate decision [6,9]. In addition, the wording of these forms should use language that is adapted to the specific characteristics of the patients to whom it is addressed so that it is easy to read and comprehension is favored [10,11,12,13,14,15,16,17,18].

Readability refers to the ease of reading a text in terms of the length of the words and sentences it contains. It is believed that the shorter the length of words and sentences, the easier it should be to read and comprehend the text. It is believed that adequate readability would facilitate comprehension and allow informed consent forms to faithfully fulfill their assigned task [11,12].

The comprehension of a text is the faculty that allows readers to understand it. Comprehension makes it possible to construct a representation of the content of the text and to integrate the information already possessed by the reader with the new information provided by the document [19].

Comprehension of informed consent forms does not depend exclusively on ease of reading or readability. Comprehension is also greatly influenced by the sociocultural and psychological characteristics of the patients to whom it is addressed. Therefore, it is necessary that informed consent forms adapt their content to the personal characteristics of the targeted patients to allow adequate comprehension, regardless of their degree of readability [20].

A study has been found that analyzes the comprehension of the informed consent process used in clinical trials using the Modular Informed Consent Comprehension Assessment (MICCA) questionnaire. This questionnaire analyzes the comprehension of the informed consent process by integrating the prior knowledge possessed by the participants with the information provided during the informed consent process. The results observed in this study indicate that only 22.3% of the clinical trial participants had a comprehension of the process that could be included within the normal range or above [21].

A study has also been located that analyzes the comprehensibility or difficulty of comprehension of informed consent forms for cardiology procedures using the Patient Education Materials Assessment Tool for Printed Materials (PEMAT-P) questionnaire. The results of this study indicate that only one of the thirty-seven forms analyzed (2.7%) reached the minimum score required to consider that the form could be comprehensible for patients, although this does not mean that it is really comprehensible, because the opinion of the patients to whom it might be addressed is not taken into account [22].

Some studies have used qualitative research techniques to analyze patients’ comprehension of informed consent forms for clinical trials. These studies have used the self-report and descriptive narrative method to assess comprehension of these forms, noting that comprehension was poor [23,24].

No questionnaire has been found to assess patients’ specific comprehension of the informed consent forms for medical or surgical procedures. Therefore, it would be interesting to develop a questionnaire that assesses the comprehension that patients really have of these forms. The results obtained with this questionnaire would make it possible to improve the informed consent forms to facilitate their comprehension and adequately help patients in making decisions in everything that affects their health. Although medical professionals in each specialty are usually responsible for completing the informed consent forms, it is often nursing professionals who frequently deliver them to patients for reading and obtain their authorization. Because of their proximity to patients and their family, nursing professionals have a primary role in the entire informed consent process and particularly in the adaptation of informed consent forms to the specific characteristics of patients to favor their comprehension [25,26]. Nursing professionals should ensure that the information contained in informed consent forms is complete and easy to read for patients [6,10]. In addition, they should check that patients have adequately comprehended all the information provided by the informed consent forms and clarify their doubts to ensure that these forms satisfactorily fulfill the ethical and legal functions entrusted to them [25,27,28].

The objective of the present study was to analyze the readability of anesthesia informed consent forms in a Spanish county hospital and to assess their comprehension by patients.

## 2. Materials and Methods

A quantitative, descriptive, cross-sectional study was carried out in patients at the Hospital de la Vega Lorenzo Guirao (Cieza, Murcia, Spain).

The study population consisted of those patients who were going to undergo surgery or endoscopic techniques and who attended the preanesthesia consultation for assessment during the month of November 2023. To select the sample, a non-probabilistic convenience sampling was performed, including all patients who met the inclusion and exclusion criteria. The inclusion criteria were being of legal age, having the capacity to answer the questionnaire, and giving oral consent to participate voluntarily in the study. The exclusion criteria were having a previously recognized sensory or cognitive disability that hinders comprehension of the informed consent form and prevents the patient from answering the questionnaire, not being fluent in Spanish, being legally incapacitated, or refusing to participate voluntarily in the study.

For the present study, the readability and comprehension by patients of the following anesthesia informed consent forms existing in our hospital were analyzed: general anesthesia (Appendix A), locoregional anesthesia, local anesthesia plus sedation, and anesthesia for endoscopies.

The INFLESZ scale was used to analyze the readability of these anesthesia informed consent forms. This scale is specifically validated to determine the readability of healthcare texts addressed to Spanish patients. Based on the length of words and sentences in the text, this scale establishes five readability grades that correspond to a given educational level and to the types of publications considered to be easily read and comprehended at each educational level (Table 1). A readability score of a healthcare text equal to or greater than 55 is considered “normal” because it can be read and comprehended by most patients [10,29].

To assess the readability of these anesthesia informed consent forms, a plain text version of each of the forms used was first made following established standards to facilitate automatic readability measurement [30]. Subsequently, the INFLESZ readability score of each of these forms was calculated using a freely available online utility [30].

In the absence of a validated tool for analyzing comprehension of the anesthesia informed consent forms, an ad hoc questionnaire designed by hospital anesthesia service personnel with more than two years of clinical and research experience was used. This questionnaire included a section on the sociodemographic data of the patients to determine age, gender, and level of education. Five levels of education were established: no education, primary education, secondary education, high school, and university studies. The questionnaire included a section where patients could indicate that they did not want to read the anesthesia informed consent form and the reason for their refusal. If patients have decided to read the form, they should answer a questionnaire with six dichotomous response criteria to assess comprehension and satisfaction with the information provided in the form (Table 2). The criteria to assess comprehension and satisfaction were scored by assigning one point to affirmative answers and zero points to negative answers, thus obtaining an overall score for each anesthesia informed consent form.

The sequence followed by the patients in the preanesthesia consultation during the collection of information was as follows. First, patients were checked to see if they met the criteria for inclusion in the study. If they could be included, they were asked if they wanted to read the corresponding anesthesia informed consent form before signing it. If they did not want to read it, they were asked their age, level of education, and the reason for their refusal. If the patients wanted to read it and participate in the study, they were given the informed consent form corresponding to the type of anesthesia they were going to undergo and the questionnaire to assess comprehension and satisfaction with the form. The patients were then transferred to a room where they could read the informed consent form calmly and carefully and complete the comprehension and satisfaction questionnaire with the information provided by these forms. Subsequently, the patients returned to the preanesthesia consultation room in case it was necessary to resolve any doubts that might have arisen during the reading of the anesthesia informed consent form.

The statistical program BM SPSS Statistics for Windows (Version 25.0. Armonk, NY, USA: IBM Corp.) was used to analyze the information obtained. For the descriptive statistical analysis of qualitative variables, frequencies and percentages were calculated, and for quantitative variables, the mean and standard deviation were calculated.

The following statistical tests were used to analyze the associations between the different variables in the study: ANOVA between polytomous nominal qualitative variable and continuous quantitative variable, Chi-square between nominal qualitative variables, Mann–Whitney U test between dichotomous nominal qualitative variable and ordinal qualitative variable, Kruskal–Wallis test between polytomous nominal qualitative variable and ordinal qualitative variable, and Student’s t test for independent samples between dichotomous nominal qualitative variable and continuous quantitative variable. Spearman’s correlation coefficient was used to analyze the relationships between discrete quantitative variables and ordinal quantitative variables. A binary logistic multivariate analysis will be performed due to the characteristics of the dependent variables.

The independent variables analyzed in this study were age, gender, level of education, and INFLENZ score of the anesthesia informed consent forms. The dependent variables are the questionnaire criteria used to analyze comprehension and patient satisfaction (Table 3).

## 3. Results

**Figure 1:**
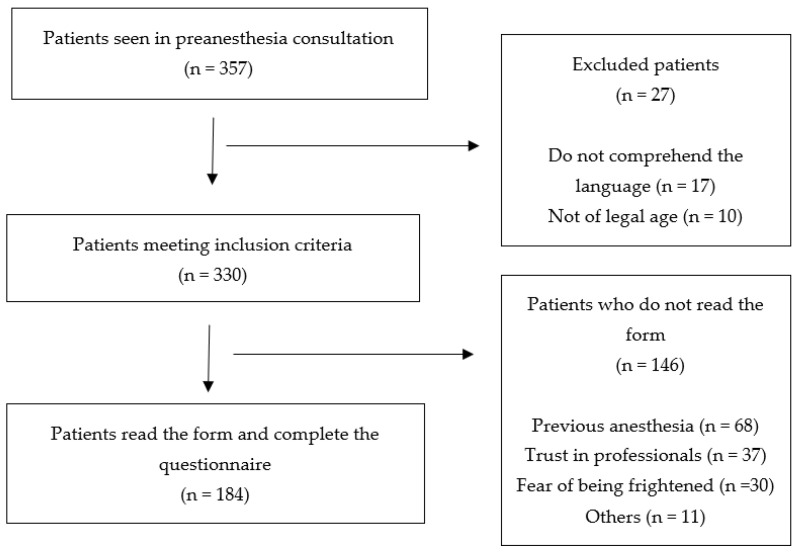
Flow diagram of participation in the study.

The patients who attended the preanesthesia consultation for assessment during the study period totaled 357. The reasons for non-inclusion were being a foreigner and not being fluent in Spanish (62.96%) and not being of legal age (37.04%). The patients who were included in the study because they met the inclusion and exclusion criteria totaled 330 (92.4%) (Figure 1). The patients who wanted to read the anesthesia informed consent form and complete the questionnaire to assess comprehension and satisfaction with the information provided totaled 184 (55.7%).

The patients included in the study had a mean age of 60.3 years with a standard deviation of 16.9 and a mode of 62 years. The age of all the participants included in the study had a graphical representation with a platykurtic distribution with negative skewness. No gender predominance was observed. More than half of these patients (58.2%) had a low cultural level (no education or primary education). The most commonly used anesthetic techniques in these patients were locoregional anesthesia (49.1%) and general anesthesia (21.8%) (Table 4).

A high percentage of patients (44.2%) decided to sign the anesthesia informed consent form without reading it. The main reason for their refusal to read the form was having previously undergone the same anesthetic technique (46.6%), followed by trust in the health professionals (25.3%) and fear of being frightened and refusing the procedure (20.5%) (Figure 1). The patients who signed without reading the form had a mean age of 63.9 years with a standard deviation of 17.3 and a mode of 78 years. The age of these patients had a graphical representation with a platykurtic distribution with negative skewness. No gender predominance was observed. Almost half of these patients (45.9%) were uneducated (Table 4). The most commonly used anesthetic techniques with these patients were locoregional anesthesia followed by general anesthesia (Table 4).

Patients who read the informed consent form before signing the anesthesia informed consent form and completed the comprehension questionnaire had a mean age of 57.5 years with a standard deviation of 16.1 and a mode of 62 years. The age of these patients had a graphical representation with a platykurtic distribution with negative skewness. No patient who read the form refused to take the comprehension questionnaire. No gender predominance was observed in this group. Almost half of these patients (47.8%) had a low level of education (no education or primary education) (Table 4). The most commonly used anesthetic techniques with these patients were locoregional anesthesia followed by general anesthesia (Table 4).

Our hospital’s four anesthesia informed consent forms analyzed in this study presented, according to the INFLESZ tool, a “somewhat difficult” readability, with a mean score of 44.8 and a standard deviation of 1.3 (Table 5).

In the descriptive analysis of the results obtained in this study by means of the questionnaire to assess comprehension of these anesthesia informed consent forms, it was observed that a high percentage of the patients who read the form and answered the questionnaire considered that the language used was not adequate (49.5%) and that they had not comprehended the form in its entirety (53.3%). Despite this, 64.1% of the patients were completely satisfied with the information received through the anesthesia informed consent form. The overall comprehension and satisfaction score with these anesthesia informed consent forms was highest for 35.9% of the patients (Table 6).

Table 7 describes the inferential statistical analysis performed between the different variables analyzed in this study, indicating the statistical test used and the statistical significance obtained.

A statistically significant negative correlation was observed between age and overall score on the questionnaire. There were significant differences according to age in the decision to read the anesthesia informed consent form, in the knowledge of the risks and alternatives of the procedure, in the appropriateness of the language used, and in the overall comprehension of the form. But no statistically significant differences were observed according to age in knowledge of the type of anesthesia to be used or in satisfaction with the information received through the anesthesia informed consent form.

According to gender, no significant differences were observed in the decision to read, neither in the overall score nor in any of the items of the questionnaire that assess comprehension and satisfaction with the information received from these anesthesia informed consent forms.

Depending on the level of studies, differences were observed in the decision to read the forms, in the overall score of the questionnaire, in the knowledge of the risks and alternatives, in the appropriateness of the language used and in the total comprehension of the anesthesia informed consent form, and in the satisfaction with the information received by these forms. No statistically significant differences were observed according to the level of education in the knowledge of the type of anesthesia to be used.

As a function of the INFLESZ readability score, statistically significant differences have been observed in the knowledge of risks and alternatives, in the appropriateness of the language used and in the total comprehension of the form, and in the satisfaction with the information received by these forms. No statistically significant differences were observed according to the INFLESZ readability score in knowledge of the type of anesthesia to be used.

A statistically significant positive correlation was observed between the INFLESZ readability score and the overall questionnaire score.

There are statistically significant differences in the INFLESZ readability score depending on the type of anesthesia informed consent form.

Multivariate analysis using binary logistic regression indicates that only patient age can predict comprehension of anesthesia informed consent forms using this questionnaire, with an odds ratio of 0.910 (95% C.I. 0.854–0.969) and a statistical significance of 0.003. The same analysis indicates that only educational level can predict patient satisfaction with these forms, with an odds ratio of 1.12 (95% C.I. 0.020–0.620).

## 4. Discussion

A high percentage of patients (44.2%) signed these anesthesia informed consent forms without reading them. The main reason for this refusal to read was having previously undergone surgery with the same anesthetic technique (46.6%), a frequent situation in an aging population with multiple pathologies such as the one analyzed in this study and one which corresponds to the type of population in industrialized countries such as Spain [31]. Trust in health professionals (25.3%) is another cause indicated for not reading the form, being common in rural areas with a low cultural level as in this case, where a personal relationship between health professionals and patients is established [32,33]. The fear of being frightened and refusing the intervention (20.5%) is another reason for not reading the forms, especially the fear of becoming aware of the risks derived from the anesthetic act to which they are going to be subjected [34]. Different studies observe that a majority of patients for different reasons adopt a passive attitude and do not read the informed consent forms so as not to have to assume the responsibility of deciding on the procedure they are going to undergo [35,36].

Although there are other tools to assess the readability of a text, the INFLESZ scale was chosen in this study to assess the readability of these anesthesia informed consent forms because it is a specific and validated tool for analyzing the ease of reading Spanish healthcare texts [29].

The anesthesia informed consent forms analyzed in this study presented a degree of readability, according to the INFLESZ scale, of “somewhat difficult”, with the form for locoregional anesthesia being the one that stands out for presenting greater reading complexity. The complexity of the wording of these forms may hinder adequate comprehension of the information they contain and hinder patients’ ability to make decisions that meet their interests and needs [13,37]. Other studies that used different tools to assess the readability of informed consent forms have obtained similar results, such that in general, these forms are difficult to read for most patients [14,15,16,17,37].

The low readability of the anesthesia informed consent forms analyzed would be in line with the degree of readability observed in specialized texts or popular science articles [29]. These forms are difficult to read and comprehend for the majority of the population, as evidenced in this study by the high percentage of patients who neither understood the language used (49.5%) nor comprehended the form in its entirety (53.3%). These results have also been observed in other studies, so it can be stated that in general, comprehension of informed consent forms is poor and it would be necessary to implement the necessary measures to improve it [21,24].

The statistically significant positive correlation observed between the INFLESZ readability score and the overall questionnaire score indicates the existence of a direct association between the readability and comprehension of these forms. There is a need to improve the readability of these anesthesia informed consent forms to promote comprehension and facilitate the informed consent process. These forms should be modified so that their readability is equal to that presented by the general and sports press that is considered “normal” on the INFLESZ scale [10,29]. To improve the readability of these forms, they should be made shorter, use short sentences, highlight the most important information, use text boxes, and even add illustrations [38,39].

The age and cultural level of these patients are determining factors in their decision to read and comprehension of anesthesia informed consent forms. Therefore, the need to modify the format and language used in these forms to adapt them to patients becomes essential in the case of older patients or those with a low cultural level. These adaptations would make it possible to motivate patients to decide to read them and would facilitate their comprehension by guaranteeing their freedom of choice [20,29,38,39].

Despite the poor readability and difficulty in comprehension of the anesthesia informed consent forms analyzed, it was observed that a high percentage of patients (64.1%) were totally satisfied with the information received. This result could be due to the fact that satisfaction is strongly influenced by other factors such as the affective support of the healthcare professional, interpersonal communication, or the motivation and expectations that patients have regarding the procedure to be performed [40,41].

To improve the readability of informed consent forms, there are several practical recommendations that can be very useful. Limit the length of the form to a single page to prevent patients from getting tired and not reading it completely. Use simple and short sentences with clear and direct language using words and concepts that are accessible to the target population, avoiding very specific health terminology. Increasing the distance between paragraphs helps readers to follow the text more easily. Place the most relevant information at the beginning of each paragraph so that patients grasp the most relevant information quickly, even if they do not read the form in its entirety. Highlighting the most important information with the use of bolding or underlining helps to direct readers’ attention to the most critical aspects of the form. Another very useful option to improve the readability of these forms could be for a person outside the healthcare profession, but with the appropriate training to understand the biomedical language used, to adapt them to the language commonly used by patients. However, it is important to keep in mind that these recommendations may not significantly influence the comprehension of informed consent forms if the specific characteristics of the intended patients are not considered. Therefore, although strategies can improve the readability of these forms, their effectiveness will depend to a large extent on how they are used in relation to the characteristics, including the cultural level or language, of the target population [5,13,36].

Adequate readability facilitates comprehension of informed consent forms, improving in practice the relationship between healthcare professionals and patients. This transparency empowers patients to make informed decisions about their healthcare, fostering their trust in healthcare professionals and improving the effectiveness and efficiency of the healthcare system. The comprehension of these forms facilitates communication with patients by serving as a basis for opening a dialogue that allows the healthcare professional to address their concerns and clarify their doubts, strengthening the relationship and reinforcing mutual respect. Good communication through comprehension of these forms and subsequent dialogue helps prevent misunderstandings, reducing the possibility of conflicts or complaints [1,2,3].

Because of their proximity to patients, nursing professionals play an essential role in adapting anesthesia informed consent forms to the characteristics and needs of patients. Nursing professionals, being in direct and continuous contact with patients, are able to detect deficiencies in the informed consent forms and help correct them to facilitate their reading and comprehension [26,42].

### Limitations

The main limitation of this study derives from the questionnaire used to assess comprehension of these anesthesia informed consent forms; this can lead to information bias. It would be necessary to improve the accuracy of the questionnaire, for example, by increasing the response possibilities for each item using a Likert scale of at least five values. To really know its internal validity, this questionnaire should be subjected to a more exhaustive validation process (test–retest stability, criterion validity, factor analysis, etc.).

Another limitation could stem from the sample selection, which might not be representative of the study population, potentially causing selection bias. For future studies, it would be advisable to conduct random sampling across the entire population to select the sample.

The external validity is limited because the study is representative of the local setting. The external validity of this questionnaire could be improved by extending the sample to other hospitals with different characteristics.

This questionnaire is a first approximation to analyze patients’ comprehension of the informed consent forms. Subsequent studies will take into account all these limitations in order to develop a validated questionnaire that will allow us to adequately analyze patients’ comprehension of these forms.

## 5. Conclusions

The anesthesia informed consent forms analyzed presented a low readability that limits their comprehension by patients. It would be necessary to improve the readability of these forms to make them easier to comprehend, especially in older patients and those with a lower level of education, and to guarantee the freedom of choice of the patients so that they can participate actively and responsibly in all aspects related to their health.

Informed consent forms, when used effectively, can significantly improve the healthcare professional–patient relationship by promoting transparency, facilitating communication, empowering the patient, increasing comprehension, encouraging active participation, reinforcing mutual respect and preventing misunderstandings, improving the effectiveness and efficiency of the healthcare system.

Some recommendations can be useful to improve the readability of informed consent forms, such as limiting their length to a single page, using simple and short sentences, not including very specific health terms, increasing the distance between paragraphs, placing the most important information at the beginning of each paragraph, highlighting the most relevant information, or adapting them by a non-health person. These recommendations may not influence the comprehension of these forms if they are not adapted to the specific characteristics of each population.

Because of the characteristics of their work, nursing professionals have an essential role to play in detecting the deficiencies in the informed consent forms and helping to correct them to facilitate their reading and improve their comprehension.

## Figures and Tables

**Table 1:** INFLESZ scale.

Score	Degree	Level of Education	Type of Publication
<40	Very difficult	University education	Scientific publishing
40–54	Somewhat difficult	Bachelor	Specialized journalism
55–64	Normal	Secondary education	General and sports publications
65–80	Easy enough	Higher primary education	Bestsellers, tabloid journalism. and tabloid press
>80	Very easy	Lower primary education	Comics

Note: García-Álvarez et al. [10].

**Table 2:** Questionnaire criteria to assess comprehension and satisfaction with the information provided by the anesthesia informed consent forms of the hospital.

Item 1. I know the type of anesthesia I am going to receive.
Item 2. I know the main risks and complications derived from the type of anesthesia I am going to receive.
Item 3. I know if the procedure can be performed with another type of anesthetic technique.
Item 4. I consider the language used in the form to be appropriate.
Item 5. I have fully comprehended the information on the form.
Item 6. I am fully satisfied with the information in the form.

**Table 3:** Characteristics of the variables used in this study.

Variable	Type
Age	Discrete quantitative variable
Gender	Dichotomous nominal qualitative variable
Level of education	Polytomous nominal qualitative variable
Comprehension and satisfaction criteria	Dichotomous nominal qualitative variable
Overall score of the questionnaire	Ordinal qualitative variable
Readability score INFLESZ	Continuous quantitative variable

**Table 4:** Summary of results. Comparison between groups.

	Total		No Reading		Yes Reading	
**Patients**	330		146 (44.2%)		188 (55.8%)	
Age						
Mean	60.3		63.9		57.5	
Mode	62		78		62	
Standard deviation	16.9		17.3		16.1	
Skewness	−0.45		−0.70		−0.34	
Kurtosis	−0.65		−0.24		−0.81	
	Frequency	Percentage	Frequency	Percentage	Frequency	Percentage
**Genre**						
Female	166	50.3	74	50.7	92	50.0
Male	164	49.7	72	49.3	92	50.0
**Level of studies**						
No studies	104	31.5	67	45.9	37	20.1
Primary education	88	26.7	37	2.3	51	27.7
Secondary education	44	13.3	17	11.6	27	14.7
Bachelor	42	12.7	10	6.8	32	17.4
University education	52	15.8	15	10.4	37	20.1
**Type of form**						
General anesthesia	72	21.8	35	24	37	20.1
Locoregional anesthesia	162	49.1	66	45.2	96	52.2
Local anesthesia plus sedation	52	15.8	25	17.1	27	14.7
Anesthesia for endoscopy	44	13.3	20	13.7	24	13.0

**Table 5:** Readability of anesthesia informed consent forms from the hospital.

Type	Syllables	Words	Phrases	Syllables/Word	Words/Phrase	INFLESZ Scale
General anesthesia	1609	705	36	2.3	19.1	45.6
Locoregional anesthesia	1503	656	30	2.3	21.2	42.9
Local anesthesia plus sedation	1194	522	26	2.9	19.3	45.0
Anesthesia for endoscopy	1609	705	36	5.4	19.1	45.6

**Table 6:** Responses to the criteria of the questionnaire to assess comprehension and satisfaction with the anesthesia informed consent forms of the hospital.

	Frequency	Percentage
Criteria: Know the type of anesthesia		
YES	178	96.7
No	6	3.3
Criteria: Know the anesthesia risks		
YES	122	66.3
No	62	33.7
Criteria: Knows the alternatives		
YES	115	62.5
No	69	37.5
Criteria: Uses appropriate language		
YES	93	50.5
No	91	49.5
Criteria: Full comprehension		
YES	86	46.7
No	98	53.3
Criteria: Satisfaction		
YES	118	64.1
No	66	35.9
Overall comprehension and satisfaction scores		
0	2	1.1
1	35	19.0
2	21	11.4
3	16	8.7
4	29	15.8
5	15	8.2
6	66	35.9

**Table 7:** Inferential statistical analysis.

Variables	Statistical Test	Statistical Significance
Age and overall questionnaire score	Spearman	0.000
Age and reading of the form	Mann Whitney U	0.000
Age and questionnaire criteria for comprehension and satisfaction analysis	ANOVA	0.001
Gender and overall score of the questionnaire	Mann Whitney U	0.762
Gender and reading of the form	Chi-square	0.902
Gender and questionnaire criteria for analyzing comprehension and satisfaction	Chi-square	0.269
Educational level and overall score of the questionnaire	Kruskal-Wallis	0.000
Educational level and reading of the questionnaire	Chi-square	0.000
Level of education and questionnaire criteria for comprehension and satisfaction analysis	Chi-square	0.000
INFLESZ score and questionnaire criteria for analyzing comprehension and satisfaction	ANOVA	0.000
INFLESZ score and overall score of the questionnaire	Spearman	0.000
INFLESZ score and type of form	ANOVA	0.000

## Data Availability

The data used to support the findings of this study are available from the corresponding author upon request.

## Public Involvement Statement

No public involvement in any aspect of this research.

## Guidelines and Standards Statement

The manuscript report conformed to STROBE guideline in the present study [43].

## Appendix A

By way of example, the informed consent form for general anesthesia is added in text form in English and Spanish.
CONSENTIMIENTO INFORMADO PARA ANESTESIA GENERAL
Yo, Don/Doña…, como paciente o representante de Don/Doña…, en pleno uso de mis facultades, libre y voluntariamente DECLARO QUE HE SIDO DEBIDAMENTE INFORMADO/A, en virtud de los derechos que marcan la LEY GENERAL DE SANIDAD Y LA LEY 41/2002 y en consecuencia, AUTORIZO al doctor/doctora… para que me sea realizada ANESTESIA GENERAL
He tenido la oportunidad de aclarar mis dudas en entrevista personal con el doctor/doctora…
Estoy satisfecho/a con la información que se me ha proporcionado (Beneficios, Riesgos, Alternativas) y entiendo que este documento puede ser REVOCADO por mí en cualquier momento, antes de la realización del procedimiento.
Y para que conste, firmo el presente documento después de leído.
Fecha y firma del/de la paciente y del doctor/doctora.
SÓLO EN CASO DE REVOCACIÓN DEL CONSENTIMIENTO
Don/Doña… con DNI… paciente/representante, no doy la autorización para la realización de esta prueba diagnóstica, o revoco el consentimiento previo, si lo hubiere otorgado. Esta decisión la tomo pese a haber sido informado suficientemente de los riesgos a que me someto al no ser sometido a esta exploración.
Fecha y firma del/de la paciente (o representante legal)
RIESGOS PERSONALIZADOS
Derivados de la situación particular de cada paciente para someterse a este procedimiento. Por mi situación actual (diabetes, obesidad, hipertensión, anemia, edad avanzada…) … puede aumentar la frecuencia o la gravedad del riesgos o complicaciones.
También me ha explicado la necesidad de advertir de mis posibles alergias medicamentosas, alteraciones de la coagulación enfermedades cardiopulmonares, existencia de prótesis, marcapasos, medicaciones actuales o cualquier otra circunstancia.
EN QUÉ CONSISTE LA ANESTESIA GENERAL
El propósito de esta información no es alarmarle ni liberar de responsabilidad al médico. Simplemente representa un esfuerzo para que usted conozca mejor los hechos y pueda tomar la decisión libre y voluntaria de autorizar o rechazar dicho procedimiento
La anestesia es un procedimiento cuya finalidad es realizar una operación sin dolor. Para anestesiarle a usted es preciso pincharle una vena por la que se le administrará los sueros y los medicamentos necesarios según su situación y tipo de cirugía prevista.
Por efecto de los fármacos anestésicos estará dormido y relajado durante la cirugía. Durante la anestesia es preciso colocarle un tubo, a través de la boca o la nariz, que llega hasta la tráquea (conducto que comunica la boca con los pulmones). Este tubo se conecta a un respirador cuya función es mantener la respiración.
Unos adhesivos con unos cables colocados en el pecho permitirán el control de su función cardiaca. El médico anestesiólogo es el encargado de controlar todo el proceso de principio a fin y tratar las posibles complicaciones que pudieran surgir.
RIESGOS TÍPICOS
Excepcionalmente, la introducción del tubo hasta la tráquea puede entrañar alguna dificultad y a pesar de hacerlo con cuidado, dañar algún diente.
Durante la colocación del tubo puede pasar al pulmón parte del contenido del estómago y ocasionar alteraciones respiratorias. Una forma de prevenir esta complicación es guardar ayuno absoluto, al menos 6 horas antes de la intervención programada. Esta complicación es seria, pero muy poco frecuente.
La administración de los “sueros” y los medicamentos que son imprescindibles durante la anestesia pueden producir excepcionalmente, reacciones alérgicas. Estas reacciones pueden llegar a ser graves, pero tienen carácter extraordinario.
Es necesario que usted sepa que las Sociedades Españolas de Anestesiología y Reanimación y de Alergología e Inmunología Clínica y los expertos de la Dirección General del Instituto Nacional de la Salud desaconsejan la práctica sistemática de pruebas de alergia a los medicamentos anestésicos, por considerar que no es adecuado hacerlo en pacientes sin historia previa de reacción adversa a los mismos, al igual que ocurre con el resto con el resto de los medicamentos. Además, estas pruebas no están libres de riesgos, y aun siendo su resultado negativo, los medicamentos probados pueden producir reacciones adversas durante el acto anestésico.
Como consecuencia de su estado clínico puede ser necesario transfundirle sangre (o algún derivado de ella) que procede de donantes sanos que no reciben ninguna compensación económica por la donación.
Cada donación es analizada con técnicas de máxima precisión para detección de determinadas enfermedades infecciosas) p. E j.: hepatitis, SIDA, etc.), que se trasmiten por la sangre. A pesar de ello la sangre y/o sus componentes pueden seguir trasmitiendo esas enfermedades, aunque con un riesgo de muy baja frecuencia.
Al igual que los medicamentos, la sangre y sus componentes pueden dar lugar a reacciones transfusionales.
Después de la anestesia, durante algunas horas, pueden aparecer algunas molestias como ronquera, náuseas y vómitos.
ALTERNATIVAS
Las alternativas a la anestesia general para este procedimiento son…
INFORMED CONSENT FOR GENERAL ANESTHESIA
I, Mr./Mrs. …, as a patient or representative of Mr./Mrs. …, in full possession of my faculties, freely and voluntarily DECLARE THAT I HAVE BEEN PROPERLY INFORMED, in accordance with the rights established by the GENERAL HEALTH LAW AND LAW 41/2002 and consequently, I AUTHORIZE Dr./Dr. … to perform GENERAL ANESTHESIA on me.
I have had the opportunity to clarify my doubts in a personal interview with Dr./Dr. …
I am satisfied with the information provided to me (Benefits, Risks, Alternatives) and understand that this document can be REVOKED by me at any time, before the procedure is performed.
And for the record, I sign this document after reading it.
Date and signature of the patient and the doctor.
ONLY IN CASE OF CONSENT REVOCATION
Mr./Mrs. … with ID …, patient/representative, do not authorize the performance of this diagnostic test, or revoke the previous consent, if granted. I make this decision despite having been sufficiently informed of the risks I undergo by not undergoing this examination.
Date and signature of the patient (or legal representative)
PERSONALIZED RISKS
Derived from the particular situation of each patient undergoing this procedure. Due to my current situation (diabetes, obesity, hypertension, anemia, advanced age…), the frequency or severity of risks or complications may increase.
I have also been explained the need to report any possible medication allergies, coagulation disorders, cardiopulmonary diseases, presence of prostheses, pacemakers, current medications, or any other circumstances.
WHAT GENERAL ANESTHESIA INVOLVES
The purpose of this information is not to alarm you or absolve the doctor of responsibility. It simply represents an effort for you to better understand the facts and make the free and voluntary decision to authorize or reject the procedure.
Anesthesia is a procedure aimed at performing a surgery painlessly. To anesthetize you, it is necessary to insert a needle into a vein through which fluids and necessary medications will be administered according to your situation and type of planned surgery.
As a result of the anesthetic drugs, you will be asleep and relaxed during the surgery. During anesthesia, it is necessary to place a tube through the mouth or nose, reaching the trachea (the passage that connects the mouth to the lungs). This tube is connected to a respirator whose function is to maintain breathing.
Adhesives with cables placed on the chest will allow monitoring of your cardiac function. The anesthesiologist is responsible for controlling the entire process from start to finish and treating any possible complications that may arise.
TYPICAL RISKS
Exceptionally, the insertion of the tube into the trachea may pose some difficulty and despite careful handling, may damage a tooth.
During tube insertion, part of the stomach contents may enter the lungs and cause respiratory disturbances. One way to prevent this complication is to fast completely for at least 6 h before the scheduled intervention. This complication is serious but very rare.
The administration of “fluids” and medications that are essential during anesthesia can exceptionally cause allergic reactions. These reactions can be severe but are extraordinary.
It is necessary for you to know that the Spanish Societies of Anesthesiology and Resuscitation and of Allergology and Clinical Immunology and the experts of the General Directorate of the National Health Institute advise against the systematic practice of allergy tests for anesthetic drugs, considering it inappropriate to do so in patients without a previous history of adverse reactions to them, as with other medications. Additionally, these tests are not without risks, and even if the result is negative, the tested medications may cause adverse reactions during the anesthesia.
Due to your clinical condition, it may be necessary to transfuse blood (or a derivative of it) from healthy donors who do not receive any financial compensation for donation.
Each donation is analyzed with maximum precision techniques to detect certain infectious diseases (e.g., hepatitis, AIDS, etc.) transmitted through blood. Despite this, blood and/or its components may still transmit these diseases, although with a very low frequency of risk.
Like medications, blood and its components can lead to transfusion reactions.
After anesthesia, for several hours, some discomfort such as hoarseness, nausea, and vomiting may occur.
ALTERNATIVES
Alternatives to general anesthesia for this procedure are…
